# RNA Interference in Practice

**DOI:** 10.1002/cfg.487

**Published:** 2005

**Authors:** Alan Lau

**Affiliations:** KuDOS Pharmaceuticals Ltd 327 Cambridge Science Park Milton Road Cambridge CB4 0WG UK


**Comparative and Functional Genomics**
*Comp Funct Genom* 2005; **6**: 320–322.
Published online in Wiley InterScience (www.interscience.wiley.com). **DOI:** 10.1002/cfg.487

### Book Review

**RNA Interference in Practice.**
By Ute Schepers. Wiley: Weinheim, Germany.
ISBN: 3527310207

In the short time since its elucidation, RNA interference (RNAi) has quickly emerged as the premier tool in which to study gene function. RNAi provides a simple and predictable yet extremely powerful and specific method in which to knock down gene expression in virtually any cell type or whole organism. The major advance in RNAi technology, particularly for mammalian systems, came with the discovery that small 21–25 nt RNA duplexes, termed ‘short interfering RNAs’ (siRNAs), mediate the gene-silencing effect. The ability to efficiently deliver silencing duplexes into cells using *in vitro* synthesized RNAs or through DNA-based expression constructs coupled with the ability to predict effective siRNA guide sequences *in silico* makes RNAi a powerful tool for systematic, genome-wide analysis of gene function. The potential of RNAi to completely revolutionize the study of gene function led to it being named ‘Breakthrough of the Year 2002’ by *Science*. RNAi-mediated gene silencing has proved to be both potent and highly specific; however, careful consideration of siRNA sequence design and experimental conditions must be taken, so that off-target effects and, in the case of mammalian systems, interferon responses are minimized. The rapid development and widespread adoption of RNAi technology means that this latest book in the *in practice* series could be a welcome addition to a lab’s manuals.

The book is divided into four chapters and begins with a comprehensive introduction defining the concepts and current knowledge regarding the molecular mechanisms of RNAi. This chapter is sub-sectioned according to pertinent questions, such as ‘miRNAs versus siRNA: two classes of small RNAs using the RNAi pathway?’, and when appropriate also tries to explain differences in mechanism of RNAi between species. It is up-to-date and is well referenced for those who would like further reading.

The remaining three chapters comprise the bulk of the book and are arranged by organism of study; *Caenorhabditis elegans, Drosophila* and mammals. The chapters on *C. elegans* and *Drosophila* are reasonably concise at around 40 pages each, whereas the mammal chapter is over 130 pages long, reflecting the additional complexity when silencing mammalian genes. Although each chapter focuses on methods specific to that organism, some protocols are universal, e.g. making siRNAs by *in vitro* transcription is described in the *C. elegans* chapter but these duplexes can also be used in *Drosophila* and mammalian cells. Each of these chapters starts with a brief introduction and is followed by an applications note, then systematically covers specific methods and protocols. Each protocol is very detailed, with step-by-step instructions and helpful technical tips or notes added in places. A list of abbreviations and a glossary of terms is also provided in the appendices. A small number of the molecular biology protocols provided are very generic, e.g. the pouring of PAGE gels, and will not be of much use to those already proficient in such applications. In addition, it is unlikely that a single lab will be using RNAi in all the organisms mentioned, so some chapters of the book may not be that relevant. However, each chapter is rich in technical detail and points to external sources of further information when appropriate, making this a useful introduction for those starting work on a particular organism for the first time. This book could also be used effectively as a general lab manual, as it covers a wide range of common methods in detail.

Chapter 2 focuses on gene silencing methods in the nematode worm *C. elegans*, a system which is very amenable to RNAi-mediated reverse genetics and useful for genome-wide screening. Protocols for *in vitro* transcription from T7/SP6/T3 promoter-driven DNA plasmid templates or by PCR using T7 promoter-linked primers to create RNA duplexes for silencing is covered here. Helpfully, the chapter also provides a short course in basic worm anatomy, propagation and microinjection techniques, as well as recommending worm

Copyright © 2005 John Wiley & Sons, Ltd.

strains for experiments. Methods for introducing silencing RNA duplexes into worms are reasonably straightforward; protocols for RNA soaking and feeding them on *E. coli* that are engineered to express dsRNA or hairpin RNAs are provided. The design and creation of both dsRNA- and hairpin RNA-expressing plasmids are described in length. Finally, the chapter closes with a brief section about genome-wide screens and suggests on-line resources for RNAi libraries.

Chapter 3 describes silencing in the fruit fly, *Drosophila*, a system traditionally useful for developmental studies. It starts by covering the applications and experimental possibilities of silencing in both *Drosophila* cell lines and adult flies. Basic methods for the creation of RNA duplexes and hairpin-RNA expression constructs, similar in principle to those for *C. elegans*, are described. *Drosophila* studies often involve the GAL4–UAS binary expression system to allow inducible and tissue-specific gene expression and their adaptation for use in gene silencing experiments is comprehensively covered. A useful table listing the advantages and disadvantages of using exogenous RNA duplexes vs. endogenously expressed hairpin-RNAs is given here and it will help in choosing the correct silencing method for your particular application. Detailed protocols for all the silencing methods mentioned are available but additional protocols for working with *Drosophila* S2 cells, as well as embryos for creation of flies, are also given. Like the previous chapter, this one finishes by briefly considering high-throughput RNAi screens and useful on-line sources of information.

Chapter 4 deals with RNAi-mediated gene silencing in mammalian systems. It is the longest chapter by far and provides a wealth of genuinely useful information on the molecular mechanisms of RNAi in addition to a comprehensive set of protocols. Unlike *C. elegans* or *Drosophila*, which typically use long RNA duplexes, RNAi-mediated silencing in mammals is normally achieved through the use of short 21–23 nt RNA duplexes, the reason for this being that mammals possess powerful antiviral or interferon-like defence pathways that become activated in response to longer double-stranded RNA and lead to global inhibition of translation and cell death. The chapter starts by explaining these facts and briefly describes how these problems were circumvented by the discovery that RNAi pathways are active in mammalian cells and that the interferon-like response can be bypassed by utilizing short RNA duplexes. The introduction of these short RNAs into mammalian cells can be done by a large variety of methods, from exogenously supplied, chemically synthesized siRNAs through to the expression of short-hairpin RNA (shRNA) from DNA-based plasmid or viral vectors. Each silencing approach, along with its variations, is systematically covered and the pros and cons of each method are clearly stated. Transient silencing methods utilizing siRNAs are dealt with first. Chemically synthesized siRNAs are widely used in gene silencing experiments and a brief overview of RNA chemistry and synthesis is given, although no protocols are provided. This is sensible, as most labs will not have access to the specialized RNA technologies that are required and siRNAs are now relatively inexpensive and widely available to buy. One key factor in successful siRNA silencing experiments is the target sequence used. The design criteria for siRNAs is very well explained and a step-by-step protocol for manually finding optimal siRNA targeting sequences is given, along with advice on how to perform homology searches for specificity. Free on-line design algorithms and companies that chemically synthesize siRNAs are also mentioned. It may be worth mentioning that siRNA design algorithms have now advanced to the point where most siRNA suppliers offer guarantees that their siRNAs will be effective, making manual designs, for most applications, unnecessary.

In a similar fashion to that described for *C. elegans* and *Drosophila*, siRNAs can be enzymatically produced through *in vitro* transcription reactions. Protocols are provided to create siRNAs from *in vitro* transcription reactions with T7 promoter-driven DNA oligos, as well as from Dicer- or RNase III-mediated digestion of long dsRNAs. Methods for the expression and purification of recombinant Dicer from baculovirus and RNase III in *E. coli* are also generously provided, although these enzymes can now be bought commercially.

Efficient delivery of siRNAs into cells is another key factor for successful gene silencing. Rather than providing an exhaustive set of transfection methods, a single protocol using a common reference procedure is instead supplied. Given the ever-increasing number of lipid-based transfection reagents available and the large number of different cell types, the reader is sensibly directed to

Copyright © 2005 John Wiley & Sons, Ltd. 

look at the suppliers' websites for further information and cell type-specific protocols. Transfection methods using the lipid-based Lipofectamine-2000 reagent and a generic electroporation protocol are described. A less well-known method of siRNA delivery is through the chemical coupling of siRNAs to cell penetration peptides (CPPs) such as Penetratin or HIV TAT sequences. Although transfection and electroporation methods of delivery are sufficient for cells in culture, they are not applicable for clinical applications and therefore CPP-coupled siRNAs may offer a solution to this problem. Methods for the chemical modification of siRNAs, coupling to CPPs and delivery into cells are all given. A small set of protocols for analysis of siRNAs by PAGE or agarose-gel electrophoresis finishes off the section about siRNAs.

Gene silencing can be also accomplished by the endogenous expression of shRNAs from DNA-based vectors. The concept and design of shRNAs are described here and methods are again based on commonly used expression systems rather than listing them all. A number of detailed methods are provided, covering RNA PolIII promoter selection, oligonucleotide cloning into DNA expression plasmids (pSUPER vectors) and the cloning, production and titration of retroviral vectors (pLentilox system). Adenoviral delivery systems are not covered. Inducible shRNA expression systems based on the tetracycline operon are discussed but no specific methods are given; instead the reader is directed to look at primary research papers. The application of RNAi silencing in mice provides another small section of discussion and methods focus on the delivery to whole mice and ES cells or oocytes. Again, the reader is referred to primary research papers for more detail.

Although the use of short RNA duplexes effectively avoids the interferon response, long hairpin RNAs (lhRNAs), similar to those typically used for *C. elegans* and *Drosophila* studies, have been found to be able to elicit an RNAi-silencing effect in some mammalian cell types. The pros and cons of using lhRNAs are defined and an in-depth series of methods follow, describing the construction of lhRNA expression plasmids using inverted repeat and direct repeat DNAs. One problem with lhRNA cloning is the high chance of recombination, and a list of suitable *E. coli* cloning strains is supplied, along with a high-throughput method to screen for full-length clones. Another problem is the possibility of interferon responses and a number of protocols are supplied with which this response can be monitored.

Like the previous chapters, discussion of RNAi in mammals finishes with a section on high-throughput silencing screens. Previously described genome-wide RNAi initiatives are discussed, with particular attention to shRNA library construction and bar-coding to identify each shRNA. RNAi microarrays are also mentioned, in which slides are imprinted with siRNA or shRNAs and cells reverse-transfected onto it. Here, the focus is clearly on shRNA-derived libraries and siRNA-derived libraries are only very briefly mentioned. The experimental considerations for actually performing library screens are not really discussed.

The book ends with a number of appendices with lists of abbreviations, protocols, RNAi reagent suppliers and an excellent glossary of terms. One minor gripe is that the appendix containing the list of protocols does not show the page numbers on which they are contained, therefore limiting its usefulness as a quick reference.

The phenomenon of RNAi-mediated 'silencing' of gene expression has become a powerful tool in the analysis of gene function and this text provides an excellent source of reference information for both experienced and novice users alike. I would recommend this book to anyone wishing to establish RNAi-mediated gene silencing in their lab.

Alan Lau

*KuDOS Pharmaceuticals Ltd, 327 Cambridge Science Park, Milton Road, Cambridge CB4 0WG, UK*

Copyright © 2005 John Wiley & Sons, Ltd. 

